# Crocin elicits potent anti-inflammatory and fibrinolytic properties post tendon injury, A new molecule for adhesion therapy^☆^

**DOI:** 10.1016/j.jtcme.2024.06.001

**Published:** 2024-06-09

**Authors:** Hamideh Naimi, Majid Khazaei, Fariba Sharifnia, Seyed Mahdi Hassanian, Sayyed-Hadi Sayyed-Hosseinian

**Affiliations:** a Department of Cellular and Molecular Biology, North Tehran Branch, Islamic Azad University, Tehran, Iran; b Metabolic syndrome Research Center, Mashhad University of Medical Sciences, Mashhad, Iran; c Department of Medical Physiology, Faculty of Medicine, Mashhad University of Medical Sciences, Mashhad, Iran; d Department of Biology, North Tehran Branch, Islamic Azad University, Tehran, Iran; e Department of Clinical Biochemistry, Faculty of Medicine, Mashhad University of Medical Sciences, Mashhad, Iran; f Orthopedic Research Center, Shahid Kamyab Hospital, Mashhad University of Medical Sciences, Mashhad, Iran

**Keywords:** Tendon adhesion, Inflammation, Crocin, Phytotherapy, Fibrinolysis

## Abstract

**Background:**

Post-surgical tendon adhesion formation is a frequent clinical complication with limited treatment options. The aim of this study is to investigate safety and efficacy of orally administration of crocin in attenuating post-operative tendon-sheath adhesion bands in an Achilles tendon rat model.

**Methods:**

Structural, mechanical, histological, and biochemical properties of Achilles tendons were analyzed in the presence and absence of crocin. Inflammation and total fibrosis of tendon tissues were graded between groups using macroscopic and histological scoring methods.

**Results:**

Crocin significantly alleviated the severity, length, and density of Achilles tendon adhesions. Moreover, the recruitment of inflammatory cells and inflammation were significantly decreased in post-operative tissue samples of the crocin-treated group, as quantified with Moran scoring system. Histological results showed that crocin elicited a potent anti-fibrotic effect on tendon tissue samples as visualized by decreasing quantity, quality, grading of fibers, and collagen deposition at the site of surgery when scored either by Tang or Ishiyama grading systems. The H&E staining showed no histo-pathological changes or damage to heart, kidney, and liver tissues of treated rats.

**Conclusion:**

Our results showed that crocin is a safe effective therapeutic candidate with potent anti-inflammatory and anti-fibrotic properties for adhesion band therapy post tendon surgery.

## Graphical abstract

**Figure:**
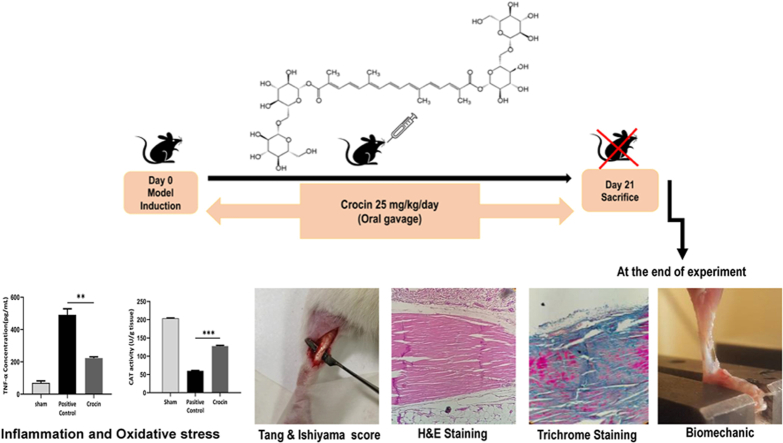

## Abbreviations

AMPK: adenosine monophosphate-activated protein kinase
CAD: coronary artery disease
CAT: catalase
COX: cyclooxygenase
CSA: cross-sectional area
DMSO: dimethylsulfoxide
DOMS: delayed-onset muscle soreness
DTNB: dithiobis-nitrobenzoic acid
EDTA: Ethylenediaminetetraacetic acid
FLS: fibroblast-like synoviocytes
H&E: Hematoxylin and Eosin
ICAM-1: intercellular adhesion molecule-1
IL-6: interleukin-6
LOX1: Lectin-like oxidized LDL receptor 1
MDA: malonyl dialdehyde
MMPs: matrix metalloproteinases
MS: multiple sclerosis
MTT: [3-(4,5-dimethyl-thiazol-2-yl5-dimethyl-thiazol-2-yl5-dimethyl-thiazol-2-yl) 2,5-diphenyl tetrazolium bromide]
NF-κB: nuclear factor kappa-B
OA: Osteoarthritis
PBS: phosphate-buffered
PCOS: polycystic ovary syndrome
PGE: prostaglandin E2
PPAR-γ: peroxisome proliferator-activated receptor γ
Saffron: Crocus sativus
SIRT1: sirtuin 1
SOD: superoxide dismutase
TBA: Thiobarbituric acid
TCA: Trichloroacetic acid
TGF-β: transforming growth factor β
TNF-α: tumor necrosis factor alpha
α-SMA: α-smooth muscle actin

## 1 Introduction

Tendon adhesions are abnormal deposits of fibrous tissue that form in the tendon area as a result of surgery. The formation of adhesion bands occurs during the tendon healing process and extends between tendon tissue and the surrounding sheath in 7–15 % of the cases undergoing tenotomy.^1^^,^^2^ Post-operative tendon adhesions represent important clinical challenges following tendon release surgeries. Tendon adhesions influence tendon gliding and lead to pain indirectly by restricting joint motion limiting recovery.^3^^,^^4^ Elevated hospitalization rate and hospital stay time, longer operative times, higher morbidity risk, and increased medical costs are adhesions-associated complications.^5^

Literature showed that a lack of balance between fibrinolytic activity and fibrin accumulation results in adhesion band formation through different processes including inflammation, coagulation, and fibrin deposition.^6^ Surgery-related injuries damage the basement membrane surfaces (epithelial or mesothelial layer) and expose them to vasculature generation and fibrin deposition recruiting fibroblasts to the damage site. In addition, infiltration of inflammatory cells from circulation as well as platelet aggregation and decreased activity of the fibrinolytic system eventually triggered the secretion of cytokines and growth factors such as transforming growth factor β (TGF-β).^6^, ^7^, ^8^ Although many efforts have been made toward clarifying the mechanisms of healing and adhesions in the tendon region, a potential solution to prevent the formation of adhesion bands remains unknown.

**Fig. 1:**
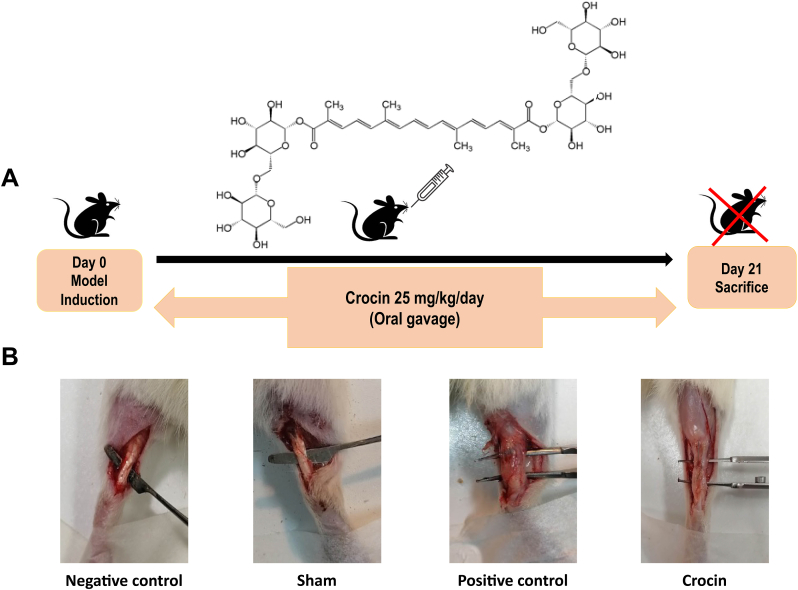
**Crocin treatment attenuated the formation of Achilles tendon adhesions.** (A) A schematic illustration of study design. (B) Compared to positive control group, adhesion band formation was reduced in the presence of crocin treatment.

Crocin is an active chemical component of Crocus sativus (saffron)^9^ with therapeutic effects on a variety of diseases.^10^ Crocin, often known as the primary biologically active component of saffron, is a class of mono- or di-glycosyl ester of the polyene dicarboxylic acid crocetin. Crocin is a water-soluble carotenoid (diterpenoid) that is principally in charge of giving stigmas their bright red hue. The crocin powder mostly consists of three crocin forms including crocin I, II, and III. Fig. 1 illustrates the crocin's chemical structure (Fig. 1).

Crocin elicits potent antioxidant,^11^ anti-inflammatory, and anti-fibrotic responses in different conditions.^12^, ^13^, ^14^ It has been shown that crocin suppresses inflammatory responses by reducing leukocyte infiltration and decreasing the mRNA expression of tumor necrosis factor-alpha (TNF-α) and intercellular adhesion molecule-1 (ICAM-1) against renal damage.^13^ Moreover, crocin administration down-regulates TGF-β, α-smooth muscle actin (α-SMA), and collagen 1a, thereby decreasing fibrotic processes in hepatic fibrosis conditions.^15^ These findings are consistent with our previously published study showing the anti-inflammatory and anti-fibrotic activities of saffron post-abdominal surgery.^16^ This study aims to examine the protective effects of crocin on decreasing adhesion band formation post-Achilles tendon surgeries a in rat model.

## 2 Material and methods

### 2.1 Materials

The pure crocin powder was obtained from Tinab Shimi Company (Mashhad, Iran). Other laboratory reagents including Phosphate buffer (PBS), Thiobarbituric acid (TBA) (CAS No.: 504-17-6), Trichloroacetic acid (TCA) (CAS No.: 76-03-9), Hydrogen peroxide (CAS No.: 7722-84-1), Ethylenediaminetetraacetic acid (EDTA), (CAS No.: 60-00-4), Dithiobis-nitrobenzoic acid (DTNB) (CAS No.: 69-78-3), Pyrogallol (CAS No.: 87-66-1) and MTT (CAS No.: 298-93-1), were purchased from Sigma-Aldrich Chemical Co., Inc. (St. Louis, MO, USA).

### 2.2 Animals

The animal experiments were conducted following the procedures outlined in the guidelines for the Care and Use of Laboratory Animals from Mashhad University of Medical Sciences. In this study, male Wistar albino rats weighing 230–250 g aged 10–12 weeks were obtained from the Laboratory Animal Center of Medical School, Mashhad University of Medical Sciences. This study was performed following the guidelines authorized by the Research Ethics Committee of Mashhad University of Medical Sciences with the ID Number “IR.MUMS.MEDICAL.REC.1400.362”.

### 2.3 The post-operational Achilles tendon adhesion model

**Fig. 2:**
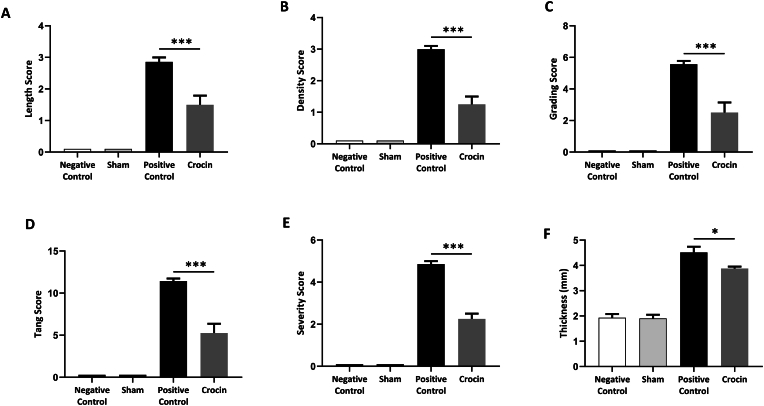
**The protective effects of crocin on macroscopic grading of Achilles tendon adhesion Bands.** (A–F) Crocin treatment showed protective effects on length (A), density (B), adhesion grade (C), overall Tang score (D), severity (E), and thickness (F) of fibrotic adhesion bands in single- or combined-treated groups. *P < 0.05, **P < 0.01, ***P < 0.001.

The model established by Tang et al.^17^ was used to induce post-operative Achilles tendon adhesions. Surgery for tendon injuries was performed under general anesthesia with an intraperitoneal injection of ketamine (15–40 mg/kg).^18^ The rat's right hind leg was shaved and prepped with povidone-iodine. A longitudinal incision (1.5–2 cm) was then made lateral to the Achilles tendon. After exposing the tendon with a surgical blade above the calcaneus insertion site, the surgical incision was closed with the Kessler-Kirchmeier technique using 4-0 polypropylene sutures. A schematic of the study design is shown in Fig. 2. Animals were randomly divided into four groups (n = 6); 1) Negative control group with no surgery and no treatment; 2) Sham group with surgical incision but no adhesion; 3) Positive control group with adhesion model induction receiving no treatment; 4) Crocin group with adhesion model induction receiving 25 mg/kg/day crocin orally for 21 days.^19^, ^20^, ^21^

### 2.4 Evaluation of adhesion scores

At the end of the experiment procedure, the right hind limb was opened through the previous incisions. Tendon adhesion tissues were graded using the Tang et al. (Table 1)^17^ and Ishiyama et al. (Table 2)^22^ adhesion scoring systems for evaluating the macroscopic properties and severity of adhesion bands, respectively. Fibrosis between groups was compared using Tang's histological grading score (microscopic), which consists of quantity, quality, and fibrosis score^17^(Table 4). After the animals were sacrificed, tissue samples were preserved in formalin (10 %) or liquid nitrogen for further histological and biochemical examinations.

**Table 1:** Tang et al. Macroscopic Grading of Achilles Tendon Adhesion Bands.

Grading		Tang et al. Macroscopic grading
**Length(quantity)**	**0** **1** **2** **3**	No adhesions <5 mm 5–10 mm >10 mm
**Density and tolerance for mobility(quality)**	**0** **1** **2** **3**	No adhesions Loose, elastic, mobile Moderate mobility Rigid, dense, immobile
**Grading of adhesions**	**0** **1 to 2** **3 to 4** **5 to 6**	Absent Inferior Medium Severe

**Table 2:** Ishiyama et al. Grading Scores for Achilles Tendon Adhesion Bands.

Grading	Ishiyama et al. grading scores
**1**	No adhesion formation
**2**	Adhesion could be separated by blunt dissection alone
**3**	Sharp dissection was needed to separate no more than 50 % of adhesion tissues
**4**	Adhesion could be separated by sharp dissection was required to separate 51–97.5 % of adhesion tissues
**5**	Sharp dissection was required to separated> 97.5 % of adhesion tissues

**Table 3:** Moran et al. Grading Scores for Inflammatory Cells Infiltration to the Injury Site.

Grading	Moran et al. scores
**0**	None
**1**	Leukocyte infiltration within fibro-osseous sheath
**2**	Infiltration of synovium and epitenon
**3**	Infiltration of endotenon
**4**	Diffuse inflammation extending within tendon and beyond sheath

**Table 4:** Tang et al. Histological Grading Scores for the Achilles Tendon Adhesion Bands.

Grading		Tang et al. Histological score
**Quantity**	**0**	No apparent adhesions
	**1**	Localized, longitudinal extension within 10 mm
	**2**	Longitudinal extension between 10 and 15 mm
	**3**	Extensive, longitudinal extension beyond 15 mm
**Quality**	**0**	No apparent adhesions
	**1**	Loose, elastic and largely movable
	**2**	Moderately dense, movable
	**3**	Dense, rigid and not movable
**Grading of adhesions**	**0**	No adhesions
	**1 to 2**	Slight adhesions
	**3 to 4**	Moderate adhesions
	**5 to 6**	Severe adhesions

### 2.5 Histological evaluations

Inflammation and fibrosis of Tendon adhesion tissues were assessed histologically using Hematoxylin and Eosin (H&E), and Masson's trichrome staining, respectively. The samples were cut into 4 μm-thick slices using a microtome. The tissues were fixed with formalin (10 %), dehydrated, embedded in paraffin, and stained with Hematoxylin-Eosin and Masson's trichrome. The influx of inflammatory cells was scored via Moran et al. (Table 3),^23^ and histological grading analysis of tendon adhesion bands was performed by a veterinary pathologist, Dr. H. S. Yazdi, using the scoring system developed by Tang et al.^17^(Table 4). The mean value from the evaluations was used based on the blinded macroscopic and histological assessments by at least two experienced individuals. Six rats were examined in each group during macroscopic evaluations. At least four slides were obtained for each rat for histological examinations, two of which were utilized for H&E and two for Masson's trichrome staining. Each slide has at least ten fields counted.

### 2.6 Oxidative stress markers analysis

The oxidant/antioxidant balance condition was analyzed using spectrophotometric methods to assess oxidative stress markers. The MDA concentrations in tissue samples were determined using the methodology of Kei et al. In summary, 2 ml of thiobarbituric acid (TBA) solution was mixed with 1 ml of 10 % tissue homogenates with boiling water and centrifuged. The absorbance was determined by using a spectrophotometer at 535 nm.^24^ The activity of the catalase enzyme was assessed by the method developed by Aebi et al. at 240 nm absorption.^25^ The total thiol content was assessed using dithiobis-nitrobenzoic acid (DTNB) reagent. SH groups and DTNB interact to produce a yellow color complex that can be read at 412 nm.^26^ The method used by Madesh resulted in the determination of SOD enzymatic activity. DMSO was used to dissolve formazan and produce a color that would last for a long time. The absorption was detected at 570 nm.^27^

### 2.7 Tendon structural and biomechanical testing

Adhesion mechanical indexes were determined as described previously.^28^^,^^29^ Briefly, following bone Achilles tendon-muscle harvesting, this complex was immersed in phosphate-buffered saline (PBS). Samples were placed on the testing machine (SANTAM-STM20) with clamps. At a crosshead speed of 5 mm/min and using a 500 N load cell, tissue was pulled to rupture (Fig. 5A). The structural and mechanical properties of tendon adhesion tissues were analyzed using ultimate load (N), ultimate stress (MPa), and elastic module (MPa) curves.^30^ The maximum load values exerted before tissue rupture are defined as the ultimate load. Ultimate stress is formulated by dividing the ultimate load value (N) by the cross-sectional area (CSA). Tangent modulus indicates the ability of specimens to resist deformation.^31^

### 2.8 Statistical analysis

Data were expressed as mean ± standard error of the mean (SEM). The GraphPad Prism software version 8 was used for data analysis and the picture analysis was performed by ImageJ software. Statistical comparisons were determined using the one-way ANOVA and Shapiro-Wilk normality test. P-values <0.05 were considered statistically significant.

## 3 Results

### 3.1 Crocin treatment potently decreased the formation of tendon adhesions

A schematic illustration of the study design is shown in Fig. 2A. On day 21, animals were sacrificed and adhesion tissues were harvested. Our clinical observation presented that gavage administration of crocin could notably attenuate the formation of post-operative Achilles tendon adhesions in rats. No adhesions were formed in the sham or negative control group (Fig. 2B).

Using Tang et al. (Table 1)^17^ and Ishiyama et al. (Table 2)^22^ scoring systems, we quantified the macroscopic characteristics and severity of adhesion bands. Our data showed that crocin significantly decreased the adhesion length (Fig. 2A), density (Fig. 2B), grading (Fig. 2C), and overall, Tang score (Fig. 2D) in the tendon adhesion model (***p < 0.001). Using the Ishiyama scoring system, we found that the severity of adhesions was significantly reduced in response to crocin treatment (Fig. 2E) (***p < 0.001). In addition, results showed that crocin attenuated the thickness of adhesion bands when compared to the positive control group (Fig. 2F) (*p < 0.05).

### 3.2 Administration of crocin reduced inflammation and oxidative stress in tendon adhesion model

**Fig. 3:**
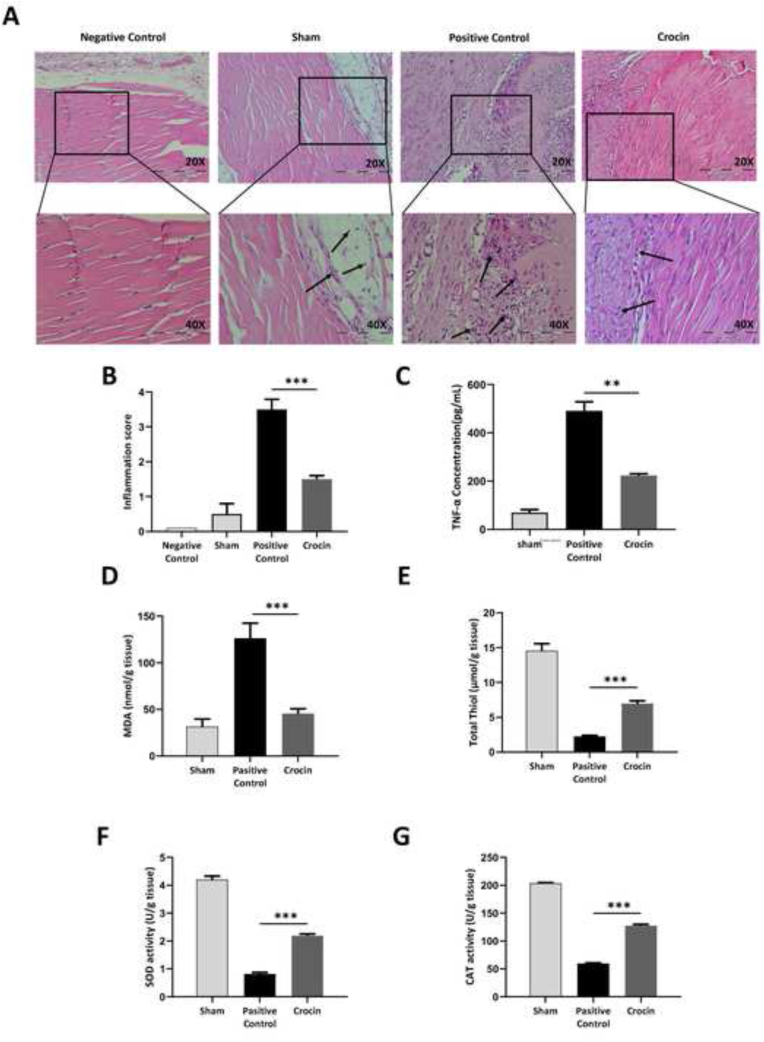
**Crocin treatment reduces morphological changes and inflammatory cell infiltration to the tendon surgery site.** (A) Administration of crocin significantly decreased inflammation in Achilles tendon tissues post-surgery. (B) Inflammation score was quantified using Moran scoring system. (C) Protein level of TNF-α was compared between different groups. Crocin administration decreased oxidative stress and (D) MDA levels and significantly increased (E) total thiol content and (F) SOD and (G) CAT activity. **P < 0.01, ***P < 0.001.

Next, we performed H&E staining to identify the protective effects of crocin on morphological changes and inflammatory cell infiltration to adhesion tissue samples. We showed that crocin treatment decreases leukocyte cell infiltration in tendon adhesion tissues. Recruitment of inflammatory cells to the tissues have been shown by arrows in the images with 40X magnification (Fig. 3A). Furthermore, a Veterinary Pathologists, quantified the histological data using the Moran et al. scoring system (Table 3).^23^ Results demonstrated that the crocin-treated group showed a significantly lower inflammation score, compared to the positive control group (Fig. 3B). Consistently, our results showed that administration of crocin significantly decreased the protein level of TNF-α in tendon tissues when compared to the positive control group (Fig. 3C). To further elucidate the effect of Crocin on oxidative stress, we measured MDA as an oxidant marker and total thiol content, SOD and CAT activity as antioxidant markers. As indicated by our results, Crocin exerted antioxidant properties and significantly decreased lipid peroxidation and MDA levels in tendon tissue homogenates. Our results also showed that Crocin exerts antioxidant effects in tendon tissue homogenates by significantly increasing the total thiol content and the activity of CAT and SOD enzymes.

### 3.3 Crocin treatment attenuated collagen accumulation and fibrosis

**Fig. 4:**
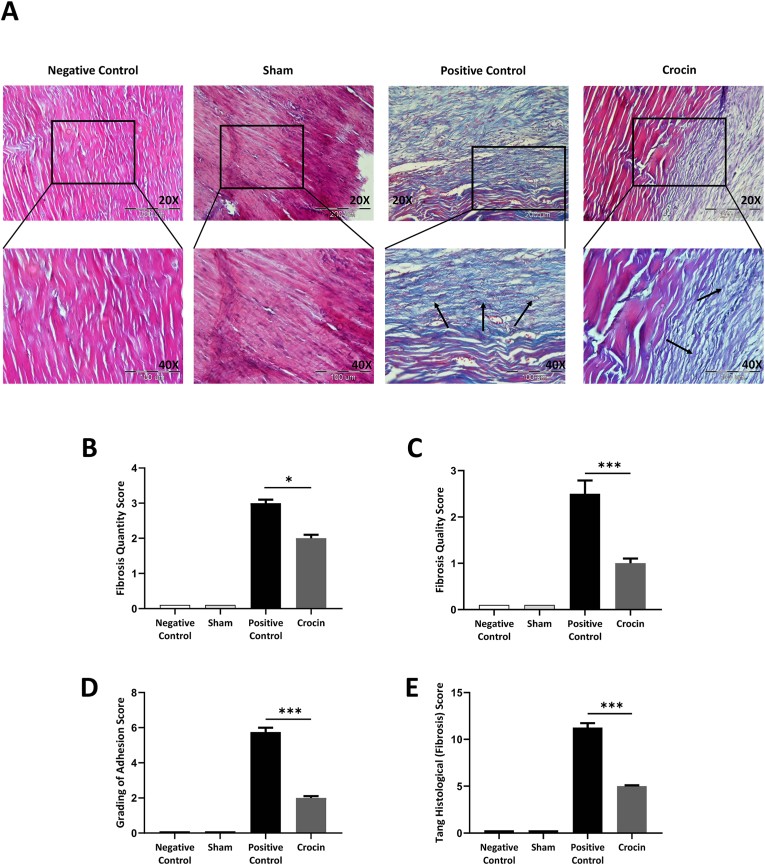
**Crocin elicits potent anti-fibrotic properties post-Achilles tendon surgery.** (A) Compared to the positive control group, gavage administration of 25 mg/kg/day crocin attenuated collagen deposition at site of surgery (Arrows indicate excessive collagen deposition). Crocin decreases (B) the quantity, (C) quality, (D) fibrosis adhesion grading, and (E) overall histological Tang score in Achilles tendon tissues. *P < 0.05, **P < 0.01, ***P < 0.001.

Next, we assessed the effects of crocin administration on fibrosis. Our results showed that treatment with crocin remarkably decreased the deposition of collagen bundle at the site of tendon injury (Fig. 4A). Deposition of collagen as well as formation of deformed fibers have been shown by arrows and compared between groups in the images with 40X magnification (Fig. 4A). A Veterinary Pathologist, quantified the histological data using the Tang et al. scoring system^17^ (Table 4). Our results showed that crocin potently decreased fibrosis quantity (Fig. 4B) and quality (Fig. 4C) scores, grading of adhesions (Fig. 4D), as well as overall histological Tang fibrosis score (Fig. 4E), supporting its potent fibrinolytic effects on tendon surgery.

**Fig. 5:**
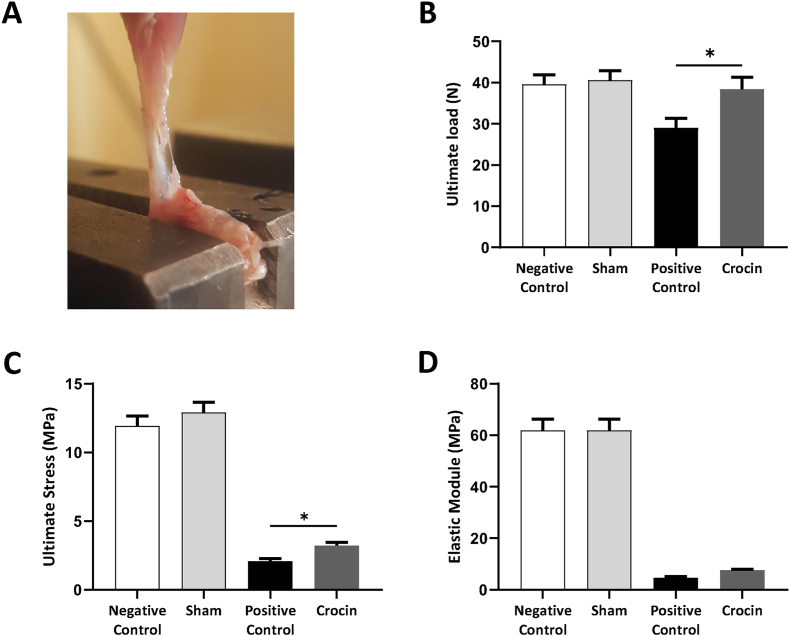
**Effects of crocin on structural and mechanical properties of injured tendons.** (A) Achilles tendon is fixed in a metal clamp in the Tensile-testing machine. (B–D) Crocin could improve the ultimate load (Fig. 5B), ultimate stress (Fig. 5C), and elastic module (Fig. 5D) in injured tendon tissues, but only the effect of crocin on ultimate load was statistically significant (*p < 0.05).

### 3.4 Effects of crocin on structural and mechanical properties of injured tendons

To investigate the effects of crocin on the biomechanical properties of injured tendons, samples were fixed in a metal clamp in the Tensile-testing machine (Fig. 5A), and ultimate load, ultimate stress, and elastic module were analyzed and compared between different groups (Table 5). Our results showed that administration of crocin improved ultimate load (Fig. 5B), ultimate stress (Fig. 5C), and the elastic module (Fig. 5D), but only the effect of crocin on ultimate load was statistically significant (*p < 0.05).

**Table 5:** Comparing structural/mechanical properties of the Achilles tendon between groups.

Group	Ultimate load (N)	Ultimate Stress (MPa)	Tangent modulus (MPa)
**Sham**	40.60 **±** 5.126	12.92 **±** 1.632	61.85 **±** 8.729
**Positive Control**	29.07 **±** 5.139	2.097 **±** 0.4806	4.622 **±** 1.257
**Crocin**	38.41 **±** 5.729	3.224 **±** 0.4674	7.651 **±** 0.6597

### 3.5 Crocin showed no toxicity-associated morphological alterations in rat organs

**Fig. 6:**
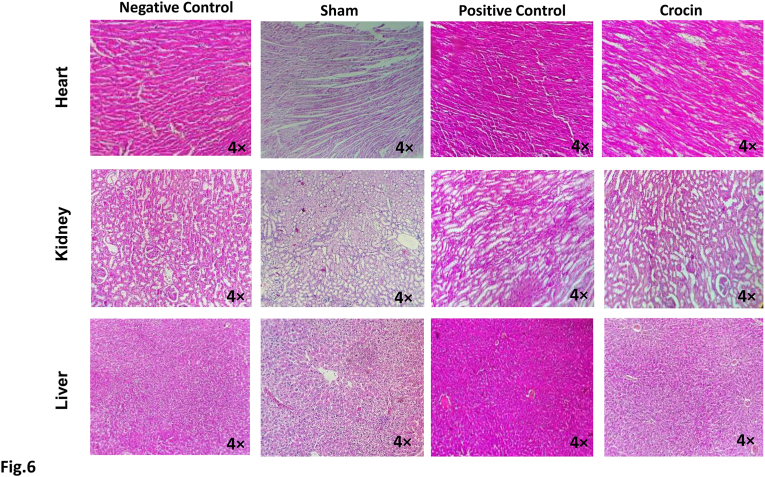
**Evaluation of crocin toxicity on heart, kidney, and liver organs.** The H&E staining showed no histo-pathological changes, infiltration of inflammatory cells to liver and kidney, nor re-arrangement of myofibers in heart in crocin-treated groups post-Achilles tendon surgery.

To investigate the crocin-related potential side effects on major organs, we assessed the heart, kidney, and liver tissues of treated rats. The H&E staining showed no histo-pathological changes or damage to these tissues (Fig. 6). Moreover, compared to the no-treatment group, no infiltration of inflammatory cells to the liver and kidney, or re-arrangement of myofibers in the heart was seen in the crocin-treated group (Fig. 6), supporting the safety of this molecule on body organs.

## 4 Discussion

In the current study, we evaluated the therapeutic effect of gavage administration of crocin on reduction of tendon adhesion formation in rat models. We showed that treatment with Crocin decreased the density, length, adhesion grading, severity, and thickness of adhesion tissues in the Achilles tendon. Consistently, our results showed that crocin reduced inflammation, oxidative stress, and collagen deposition whereas improved tendon biomechanical properties at the site of tendon surgery.

Given the safety and therapeutic effects of Crocin, various research teams previously investigated the protective effects of Crocin against disorders related to bones, muscles, and tendons. Using an experimental model of gastrocnemius muscle ischemia/reperfusion injury in the rat, Yildirim et al., 2023 demonstrated that Crocin protected gastrocnemius muscle from IR injury by mediating the expression of inflammatory markers including TNF-α, IL-1β, and IL-6, and stimulating the activity of antioxidant agents like CAT, SOD, and GPx.^32^ Moreover, Lei et al., 2017 also showed that Crocin-mediated Osteoarthritis-related symptoms and joint pain via alleviating the IL-6 levels and Oxidative stress in meniscectomy (MNX) surgery-induced osteoarthritis model in rats.^33^ Ding et al., 2013 showed the protective effects of Crocin against cartilage degeneration in an Osteoarthritis-induced model of cartilage degeneration in rabbits. They found decreased cartilage degeneration and IL-1β levels in the knee joint post-crocin injection.^34^ Furthermore, Li et al., 2015 in their study investigated the anti-inflammatory properties of Crocin in rats’ intervertebral discs. They showed that treatment of isolated nucleus pulposus cells with Crocin decreased the inflammatory response as indicated by a reduction in TNF-α, IL-1β, and IL-6 levels and inhibition of MAPK and JNK pathways.^35^ In a rat model of Rheumatoid Arthritis, Hu et al., 2019 showed that crocin injection decreased RA-associated symptoms like paw swelling which was accompanied by a reduction in TNF-α and TGF-β levels.^36^ Similarly, in a study conducted by Li et al., 2018, crocin administration in fibroblast-like synoviocytes (FLS) of RA-induced mice caused a reduction in TNF-α, IL-1β and IL-6 levels, in-vivo.^37^ Hemshekhar et al., 2012 showed that crocin can reduce the serum concentration of inflammatory factors including tumor necrosis factor-alpha (TNF-α), interleukin-6 (IL-6), interleukin-1β (IL-1β), NF-κB, ROS, cyclooxygenase (COX)-2, and prostaglandin E2 (PGE) as well as matrix metalloproteinases (MMPs)-3, MMP-9, MMP-13 in arthritis model.^38^

Inflammation in the injured tendon usually triggers ROS production and oxidative stress which causes damage to the tendon tissue as it exceeds the antioxidant capacity of the tissue cells.^39^ Studies showed that some inflammatory mediators including IL-1β and TNF-α stimulate ROS production in the tendon cells, aggravating the tendon damage.^40^ Previously, increased oxidative stress, ROS content, and MDA levels, and decreased SOD activity and GSH content were reported in animal models.^41^^,^^42^ Previous studies showed that Crocin regulates oxidative stress through the elimination of free radicals and stimulation of the anti-oxidative enzymes’ activity such as SOD, GPx, and CAT. The MDA levels and the amount of peroxidized membrane lipids were shown to decrease by crocin.^43^^,^^44^ Consistent with the literature, we showed decreased oxidative stress and MDA levels, increased antioxidant activity, enhanced SOD and CAT activity, and increased total thiol content in the tendon tissue of the crocin-treated animals.

There are several studies supporting the therapeutic effects of crocin on inflammatory- and fibrotic-associated diseases.^45^^,^^46^ Samarghandian et al., 2016 showed the protective effects of crocin against oxidative stress and inflammation in aged rat kidneys.^47^ In line with these results, the protective activities of crocin have been investigated in breast cancer studies conducted by Hashemi et al., 2020 representing a lower activity of catalase and superoxide dismutase in crocin-treated mice.^48^^,^^49^ The benefits of crocin treatment were also clinically investigated in patients with coronary artery disease (CAD). Crocin significantly enhanced the expression of adenosine monophosphate-activated protein kinase (AMPK) and sirtuin 1 (SIRT1), while decreasing Lectin-like oxidized LDL receptor 1 (LOX1) and NF-κB in mRNA levels.^50^ Algandaby et al., 2018 showed the anti-fibrotic/inflammatory function of crocin in thioacetamide-induced liver fibrosis.^51^ In addition, Chhimwal et al., 2020 showed that crocin induced the expression of peroxisome proliferator-activated receptor γ (PPAR-γ) inhibiting the fibrogenic activity and fibrosis in CCl4-exposed rats.^52^ Moreover, we recently investigated the effects of crocin administration on the ulcerative colitis mice model. The crocin-treated mice presented less diarrhea, rectal bleeding, and body weight loss. Crocin also improved the morphological alterations and reduced inflammatory responses and fibrogenesis,^53^ which was consistent with our recent manuscript on post-operative peritoneal fibrotic bands.^54^ Consistently, in this study, we showed that suppression of inflammation and fibrosis are two key players in eliciting protective properties of crocin in decreasing adhesion band formation post Achilles tendon surgery.

The biomechanical outcomes of the current investigation also provided additional support for enhanced tissue quality with crocin therapy. Mechanical testing revealed that, as compared to the control group, crocin considerably increased the ultimate stress and Young's modulus of the regenerated tendon tissues.

In terms of the clinical application of crocin, multiple clinical studies investigated the therapeutic potency of crocin in different disorders. In Osteoarthritis (OA) patients, Krocina™ exerts immunomodulatory effects by reducing the pro-inflammatory cytokine, IL-17. Similarly, Mohebbi et al., 2022 showed that Crocin modulated inflammation in OA patients by stimulating the expression of MiRs responsible for regulating inflammation.^55^ A 10-day oral Crocin supplementation also showed protective effects against Delayed-onset muscle soreness (DOMS) in patients and significantly enhanced clinical outcomes.^56^ Furthermore, Crocin supplementation showed protective effects in the treatment of polycystic ovary syndrome (PCOS), as Crocin supplementation decreased the concentration of inflammatory markers including TNF-α and IL-6.^57^ In another clinical study on multiple sclerosis (MS) patients, 4 weeks of Crocin treatment induced a significant reduction in the levels of important pathogenic factors in MS, including lipid peroxidation, DNA damage, serum TNF-α, and IL-17 levels.^58^

Taken together, these findings suggest that crocin could attenuate the Achilles tendon adhesion formation, at least partially by decreasing inflammation, oxidative stress, and collagen accumulation in damaged tissues. Further animal and clinical studies are needed to better clarify mechanisms underlying the anti-inflammatory and anti-fibrotic properties of crocin and validate the therapeutic potency of crocin as the main therapy or in combination with the standard therapeutics in decreasing complications of post-tendon surgeries in patients.

A potential limitation of this study is that we were not able to evaluate the effect of crocin on the transcriptional expression of the inflammation and fibrosis-related genes because of the tendon tissue nature and its high collagen content which significantly interfered with the RNA extraction process.

## Author's contributions

H. N. designed and performed cellular, molecular, and animal experiments. F. S. discussed the results and contributed to the final manuscript. S. S. analyzed data and contributed to the clinical interpretation of the results. S. M. H. and M. K. designed the study plan and supervised the project.

## Declaration of competing interest

The authors have no conflicts of interest.
